# Harnessing the sustainable competitive advantage of social motivation in the informal market: A West African society insight

**DOI:** 10.1016/j.heliyon.2021.e07538

**Published:** 2021-07-10

**Authors:** Adebanji William Ayeni, Olaleke Ogunnaike, Edidiong Ayeni, Oluwole Iyiola

**Affiliations:** a Department of Business Studies, Landmark University, Nigeria; b Department of Business Management, Covenant University, Nigeria; c Department of Political Science, Landmark University, Nigeria; d Landmark University SDG 1 (No Poverty), Nigeria; e Landmark University SDG 5 (Gender Equality Research Group), Nigeria; f Landmark University SDG 8 (Economic Growth Research Group), Nigeria

**Keywords:** Informal entrepreneurship, Social motivation, Sustainable competitive advantage

## Abstract

The existence of informal entrepreneurship is alleged to curb hardships related to unemployment. This has been the case of Nigerian youths at getting needs met but a dire exists when related to the African cultural heritage of value and goals. The study examines social motivations of informal entrepreneurs and their implications for business performance in selected electronics markets with the engagement of sustainable competitive advantage in southwest of Nigeria. Causal research design was deemed appropriate. The survey design was integrated to describe the present trend in the informal electronic market. The need to obtain subjective opinion of the respondents and draw an accurate assessment of the entire population via the studied sample calls for the adoption of the descriptive survey research design. Using linear regression, it was revealed that social motivation has a statistical significance in predicting sustainable competitive advantage recording the beta value of (beta = 0.389 with t-val (9.822) higher than 1.96, sig.000 p < .05). The study reflected that social motivation makes a strong contribution to explaining sustainable competitive advantage in the informal market.

## 1 Introduction

The West African Society is perceived to be characterised by materialism, the degree of social acceptance of an individual, being a direct function of the person's economic power. In such a society, people desire to gain social acceptance, respect and recognition by enhancing their economic status through informal entrepreneurship (Elbahnasawy et al., 2016). In other words, some persons are socially motivated to become entrepreneurs, though informally to express and feel their loved by the same society. This human need was well articulated by Maslow (1956) in his third stage of the hierarchy of needs (Badenhorst-Weiss et al., 2014).

Therefore, the rise in socially motivated entrepreneurs, either formal or informal, is what gave birth to the branch of entrepreneurship called social entrepreneurship (Amir et al., 2016). Social entrepreneurship is the use of business models in meeting the social needs (expression of love to a society) of a society (Aquino et al., 2018). Thus, the existence of informal entrepreneurship is alleged to curb hardships related to unemployment. This has been the case of Nigerian youths at getting needs met but a dire exists when related to the African cultural heritage of value and goals attainment against the witnessed occurrence (Maina, 2013; Ayeni et al., 2019). Furthermore, Block's et al. (2014) the attainment of success on the global stage is inclined to correct the irony of age with success as the 21^st^-century notable world revolutionaries have gotten the expected glorified stage at their respective youthful age. This positive attainment has started a drive within the Nigerian system towards a negative attitude of ‘get rich syndrome’, thereby whitewashing the established values the Nigerian culture portrayed before now towards wealth attainment (Yagboyaju, 2017; Adebanji et al., 2018).

In line with the above discussion, the study hypothesizes that;

H_o_: Social motivation does not have a significant effect on sustainable competitive advantage in the informal market.

On this premise, the study examines social motivations of informal entrepreneurs and their implications for business performance in selected electronics markets with the engagement of sustainable competitive advantage in southwest of Nigeria.

### 1.1 Makeup of motivation process

Roy and Wheeler (2006) postulate that motivating process begins when an unsatisfied inner desire or weakness that an individual has is identified or recognized. For example, a person may feel the need for promotion or higher income, food, companionship or development. An internal state is required, which causes friction in someone who goes in a certain direction to seek ways to fulfil that need. After recognizing the unmet need and considering various ways of meeting it, one of the ways of meeting the need is chosen (goal-direction behaviour). Tension vanishes as requirements are met. This cycle is repeated on various platforms with the attainment of the same result of satisfaction. The position of Ogunrinola (1991) posited that for an informal entrepreneur, the perception of continuous engagement in the market as with the established aghast laws again can only come at the birth of a drive. The next session highlights this.

### 1.2 Social motivations of informal entrepreneurs

Social motivation involves the drive to attain social value by the informal entrepreneur as the participation within the informal business will enable enhancement of the entrepreneur's social status. It further includes the provision of a platform for social incentives such as catering for the entrepreneur and the entrepreneur's family. Anetor (2015) stresses the opinion that the influence of social motivation which maintains the informal business operation was created to preserve the interpersonal relationships with the entrepreneur's colleagues. Thus, the entrepreneur's involvement in the informal market enables the establishment, maintenance, and restoration of positive affective relations with others. According to Douglas and Prentice (2019), social motivation births cordial relation with other informal entrepreneurs which encourages the entrepreneurs' willingness to operate informally.

Dagmar (2014) views McClelland's motivation needs theory as predicated on the studies of managers, having three most important needs, namely: achievement, affiliation, and power. The achievement segment associates the needs theory with the requirement for competitive success measured against a personal standard of excellence. The affiliation segment was focused on the need for warm, friendly relationships with others through interpersonal relationships. The power segment is in sync with the search for the ability and desire to control and influence others. This can be summed as social motivation. Collectively, social and economic goals are achieved via the chosen means of business operation utilised (Webb et al., 2013). In sociology, motivation studies have emphasized the relationship between socially-oriented situational factors that push individuals to act outside the laws and regulatory guidelines in society (Godfrey, 2011).

One of the established goals of entrepreneurship since its inception is wealth creation while the creation of value has also been found to be of necessity for participating entrepreneurs. In collectiveness, a social and economic goal are achieved via the chosen means of business operation indulge in (Webb et al., 2013). In sociology, motivation studies have emphasized the relationship between socially-oriented situational factors that push individuals to act outside the laws and regulatory guidelines in society (Minola et al., 2016). This act is seen as a symptom of disassociation between the structures that have been built for a society due to the lack of utilisation of culturally prescribed aspirations in the realization of those desires.

Merton (1968) further gave reasons why such an identified strain occurs which limits inaccessibility through a legitimate means of the said individual in achieving the predesigned societal goals (e.g., monetary success). It has been noted by State (2014) that the Nigerian societal goals have changed in accordance with the political instability structure, leading to the absorption of a new culture. Invariably, in most economies, an individual in such a need will attempt to use illegitimate means to get his desires with a constant reminder of the new absorbed culture. This had been worsened by the media via the creation of *aide memoire* from the public wealth, status, ownership and visible assets of others, thereby creating a motivator (in its sense, a strain) to the business practitioners operating in the informal sector. This was further established by Fadayomi and Olurinola (2015) who reflected on the positive relationship between income gap and informality as against the negative association between income level and informality. The assumption created is that the means through which opportunities are achieved are in less relevance when compared with the value creation of an entrepreneur. Iyiola and Azuh (2014) also share the same views. On this premise, Benzing and Chu (2005) states that the process theory of motivation argues that motivating people is a rational internal cognition process rather than an external process. This process approaches are not particularly concerned with the needs of people but with the decision-making process through which motivation takes place. This can be skewed towards the available opportunity present in the informal sector as regards this study. In lieu of this, there is the ability to determine what lines up the drive toward the operation in the economy. The form of motivation of the informal entrepreneurs established by this study is social motivation. It is on this premise that the next section discusses the identity status theory to establish the link between social motivations that promotes entrepreneurship.

### 1.3 Understanding the identity status theory

Refining and extending Erik Erikson's work of 1968 on the development of coherent sense of identity of adolescence, James Marcia (1991, 1994, 1999, 2002) came up with four Identity Statuses of psychological identity development. The main idea was that one's sense of identity is determined largely by the choices and commitments made regarding certain personal and social traits. The theory came from the ground-breaking work of Erik Erikson on identity and psychosocial development (Marcia, 1966). James Marcia, being a developmental psychologist, refined and extended Erikson's model, primarily focusing on adolescent development. He decided to address the thought of Erikson's identity challenge by saying that the teens' stage is not a response to identity or a confusion of identity. However, the degree to which one explores and engages in an identity in different areas of life, from the fields of vocation, sexuality, gender related choices, among many others. Identity achievement theory of Marcia proposes that two separate pieces shape an identity of a teenager: crisis and dedication. This thesis considers Crisis as a moment of revolution in which old ideals or decisions are reviewed. The final product of a crisis is an undertaking. This is further made into certain roles or values. Having developed a semi-structured interview for identity research, Marcia recommended identity statuses of psychological identity development. These posited statuses are: identity diffusion, identity foreclosure, identity moratorium and identity achievement. It must be noted that the statuses are not stages and should not be viewed as a sequential process. Identity crisis takes the shape of subsequent adult life cycle phases and different life experiences. Depending on the individual, real life events including a loved one's death, work loss, changing and other events can cause imbalances. This is only valid, however, when a person has established some sort of identification. Diffusions remain static and attempts to create an identity have not been made and so no identity can be reformed. Many would opt to live in an atmosphere close to their childhood memories with foreclosures in order to stay untouched. Thus, if the life of forecasting is unbalanced, it may have special consequences. This can lead to the beginning of a period of re-construction, also known as the moratorium-achievement-moratorium-achievement (MAMA) cycles (Meeus, 2011).

### 1.4 Criticism of Marcia's theory of identity status

It must be acknowledged that Marcia's theory of identity aided in describing the origin and development of diverse identity statuses though with limitations. According to Baerveldt et al. (2003), the theory's main oversight is that it is primarily focused on the late adolescent years. The theory is applicable in later adulthood, when identity crises may reoccur. At this stage, it is difficult to make a suggestible turnaround in the individual life, making it an observatory and hardly implementable study to effect a change.

Hoegh and Bourgeois (2002) reveal that more than three decades earlier, a thorough study of identity literature by Marcia (1980) demonstrated that attainment of identity (and moratorium to a lesser extent) was correlated with generally thought to be attractive psychological variables. In the other hand, diffusion of personality (and a lesser extent foreclosure) appeared to be correlated with standard unintended psychological variables. Over the years, reviews of a similar kind focussed on various variables have concluded that identity achievement has the most adaptive benefit, whereas identity diffusion is most harmful to relevant facets of psychological work, learning and development. This was with moratorium being the second most beneficial and foreclosure being the second most damaging. Megreya and Ahmed (2011) examine the identification status of Egyptian-Kuwaiti students in Middle East countries, including examining cross-cultural gaps. The researchers were surprised that the initial theory of Marcia did not pay much attention to and tried to broaden the cultural context. In terms of the four different status, men had more foreclosures than women, while Egypt had more performances, fewer foreclosures and less spread than Kuwaitis. Also, in roughly related Middle Eastern cultures, these distinctions seem to indicate that both macro and cultural environments are heavily influenced by identification development. The work of Crocetti et al. (2008) explored the correlations between the identity statuses of Marcia's model and social behaviours reveal that it was focused on young adults ranging in age from 19 to 35 years of age. From the findings, the scholars made it known that people's identity status is not specifically limited to an age group as individuals may explore elements tied to their identity throughout life. Such elements include faith, ideology, and occupational preference to name a few. Cheng (2004) compares Taiwan's identity formation to that of the USA. There are striking social disparities in both cultures, whereas Taiwan is a collectivist society while the United States represents an individualistic culture. These variations were supported by findings and their important impact on identity development in both cultures was investigated. Gender has played an important role and served as a reason for societal ambiguity. This influence can be emphasized when comparing western culture's identity forming process with that of a non-western one. In the loopholes suggested, Hardy and Kisling (2006), among others, note that theoretical identification status seems to be suited to various cultures in a versatile manner. There appears to be a lot of cross-cultural confirmation of identification statuses suggested by Marcia and, in addition, cultural variations tend to influence identity status.

### 1.5 Relevance of the theory to the study

The theory provides the necessary framework for achieving the objective of the study. The objective of determining the effect of social motivation on sustainable competitive advantage in the informal market. Marcia's theory of identity achievement relates that two distinct parts form an adolescent's identity: crisis and commitment to the social drive of achievement. This was shown by relating the informal entrepreneurs' complexities of engagement in the electronics market to their respective status identity or vice-versa. Furthermore, the theory is able to link the existence of informal entrepreneurship to curbing hardships related to unemployment. This has been the case of the Nigerian youth at getting needs met even when a dire exists as against the expected African cultural heritage of value and goals attainment. Thus, the theory shows how the attainment of success on the global stage is inclined to correct the irony of age with success as the 21st century notable world revolutionaries have got the expected glorified stage at their respective youthful age.

## 2 Methodology

The conducted study is a quantitative and causal in nature. The study was conducted in six markets in six south-western states. These include Lagos, Ogun, Oyo, Osun, Ondo and Ekiti States. The markets considered for the research were Ikeja computer village, Okelewo market, Bola Ige International market, Fagbesa market, Olukayode shopping complex and Ayo Fayose market respectively. They were analysed by periodic sighting which was carried out thrice with the inclusion of Saturday, a busy day at the various markets. The estimation for each location is:

i Ikeja Computer Village has three heavily populated streets of informal business entrepreneurs. The initial two populated streets, Otigba and Ola Ayeni had 245 and 164 sighted entrepreneurs respectively summed up to 409, averaged at 204.5, and approximated to 205. There are 11 streets in Computer Village, Ikeja and they are: 1. Otigba Street, 2. Francis Oremeji, Street, 3. Ola Ayeni Street, 4. Kodesho Street, 5. Simbiat Abiola Way, 6. Obafemi Awolowo Way, 7. Oshitelu Street, 8. Idowu Lane, 9. Adepele Street, 10. Peeple Street, 11. Osundairo Street. With the listed named streets, the averaged 205 informal entrepreneurs were multiplied by the 11 streets to get the population of the entrepreneurs operating in the informal and this leads to 2255.
ii In Ondo State, Olukayode Shopping Complex in Akure was the chosen market for the study. It has four operating floors with the entrance utilised for business purpose. The informal electronics entrepreneurs were also found conducting business activities along the following streets with Olukayode Shopping Complex being in the middle: 1. Igbataye street, 2. Moferere Street, 3. Oba Adesida Road. The population for the 4 floors were estimated as: floor 1 (82), floor 2 (63), floor 3(25) and floor 4 (32). The utilised frontage had 47 operating informal entrepreneurs. The streets utilized for business around the complex surrounding had: Igbataye Street (78), Moferere Street (32) and Oba Adesida Road (115). With the above listed locations in the market, the summation of the informal entrepreneurs in the Olukayode Market were estimated at 474.
iii In Oyo state, the assessed market was Bola Ige International market. The market is shielded by Ibadan-Ife Road and Old Oyo Road and an hotel. There was no constructed pathway except those made by human trade path and these are the means of accessibility within the market. The pathways are roughly parted via human terracing. They are no named lanes. The front area of the market facing Ibadan-Ife road had a total of 148 informal entrepreneurs operating under umbrellas while the back of the market had 135 informal entrepreneurs using themselves as the shop or are under the roof coverings of the main stores. In the market, there were a total of eight easily accessible pathways and other hard to access ways but that are well known to the operators. From the calculated entrepreneurs of 283, an average of 142 (141.5 approximated) entrepreneurs culminate to an approximation of 1136 informal electronics entrepreneurs in Bola Ige International market.
iv For Ogun state, Okelewo market was chosen for the study. The location was an abandon government building project with six floors. Only the ground floor was utilised for business operation. They were roughly scattered around the terrace of the building with the use of the umbrella stand to showcase the products or tools designated for repairs. Outside the street, a total of 227 were operating around the location while 89 were across the road. It was noticed that there was a high level of mobility with the entrepreneurs and to this end, the products were noted to be quite handy as to be carted by them. In the uncompleted structured, 356 entrepreneurs were noted to be operating either under a stand or operating in pairs of two or four. With the above listed locations, within and outside the market, the summation of the informal entrepreneurs in the Okelewo market is estimated at 672.
v In Ekiti State, the Ayo Fayose market was chosen. It is a2 hectares with 150 buildings and a car park located at Ijibo roundabout beside Total Filling Station. In the market, electronics shops were well sited with three major points serving as exit and entry with all shops erected in bungalow shaped buildings. The summation of the informal entrepreneurs at the Ayo Fayose market is estimated at 334.
vi In Osun State, Fagbesa Market is located around a saturated residential/business location. The presence of heavy traffic around the junction makes it quite a niche for the informal market. It is a street which was residential but turned to a market. The single lane leads to Atakumosa Market and Government office, Osogbo. The estimated informal entrepreneurs were placed at 427.

The selected markets totalled an estimated sum of 5298 and were made of Lagos - Computer Village (2255), Ondo - Olukayode Complex (474), Oyo - Bola Ige International market (1136); Ekiti - Ayo Fayose market (334), Ogun - Okelewo market (672) and Osun- Fagbese Adenle (427). Based on this summation, the average was calculated against the determined 600 sample size.

In addition, causal research design was deemed appropriate as the study sought to examine the implication of social motivations of informal entrepreneurs for business performance in selected electronics markets with the engagement of sustainable competitive advantage. For the cause of the study, the independent variable was deemed to be social motivation while the dependent variable was sustainable competitive advantage.

The survey design was integrated to describe the present trend in the informal electronic market transition to formal business practices enhanced from the perspective of motivation. The need to obtain subjective opinion of the respondents and draw an accurate assessment of the entire population via the studied sample calls for the adoption of the descriptive survey research design (Osuala, 2005). With the end goal of this research, the positivist paradigm was utilized because of the accompanying reasons. The investigation plans to test a conceptual framework that requires the definition and trial of hypotheses utilizing statistical strategies to dissect the information so as to arrive at a logical result. Additionally, there is a need for a profoundly organized strategy to guarantee unwavering quality and replication of the exploration and to take into consideration the comparison of its discoveries (Datta, 2010).

Due to the large number of informal entrepreneurs situated in the listed electronic markets, it was deduced that the population of informal entrepreneurs are infinite as it was noted that they change places of business operations and are mostly mobile. This assertion came from the observation of entrepreneur's inconsistency where they conduct business transactions. An informal entrepreneur in any of the listed market was observed to own a stand paid for daily. This was located by the street/road side. To arrive at the sample size of an infinite population, Kumar (2012) suggested a formula for deriving it as:

SS = Sample Size

Z = Given z value

p = Percentage of Population

C = Confidence level

P = Population

Therefore:

$$\text{SS}=\frac{{\text{Z}}^{2}\text{p}\left(1-\text{P}\right)}{{\text{C}}^{2}}$$

With the above formula, the sample size in its infinite population will be calculated as thus

$$\text{N}=\frac{{\left(1.96\right)}^{2}\left(0.5\right)\left(0.5\right)}{{\left(0.04\right)}^{2}}=600.25\;\sum 600\;\text{Respondents}$$

z- The normal deviate corresponding to the desire confidence level = 1.96

p-is the estimate proportion of an attribute that is present in the population = 0.5

q-the opposite of p, q = 1-p = 0.5,

d-Degree of accuracy desired = 0.04

n-Minimum sample size.

This agrees with Krejcie and Morgan (1970) on determining sampling size of five hundred as the appropriate unit for acceptable paradigm rationality in a large population scenario. The Link-tracing method was used to study the hard-to-reach populations, by engaging respondents that can access the market, also known as gatekeepers. These gatekeepers extended their reach by recruiting their peers. These increasing peers, accrued from the recruitment exercise, are termed as snowball sampling. Thus, the adopted quasi-percentile for the study is a mixture of the snowball and link tracing sampling method in accessing the population. The adoptions called for the use of a gatekeeper to get honest opinions of the respondents in the interested market. Due to the nature of the business being hidden with a compulsory protective access to information being hard, a gatekeeper, holding the accessibility in terms of control of power and authority, was required. Therefore, with these the infinite formula in defining the size to be measured for the study, the arrived sample size of 600 was distributed and shared in the listed markets for data collection. Thus, to ensure an efficient distribution among the stated markets that are not equally patronised on the basis of the volume of population and easy access to knowledgeable respondents about the study's intent, the snowball was utilised Adopting the quasi percentile for the market location analyses called for a periodic sighting to get an estimated number of informal within the market.

### 2.1 Ethics

Via one-on-one interactions with the research assistants, participants were given verbal knowledge about the analysis. All participants were told that the study was completely voluntary, that they could leave at any time without explanation, and that their privacy would be protected. By filling out the questionnaire, the participants decided to participate. Furthermore, when it comes to the informal electronics industry in, it is not customary to obtain the permission of a small group of people to elicit responses to research questions in Nigeria. Less emphasis is attached to ethics on research that bother less or with no connection with health matters (see Table 1).

**Table 1:** Distribution of questionnaires to selected markets.

S/N	Market Names	States	Quantitative samplings
1	Computer Village, Ikeja	Lagos	255
2	Bola Ige International Market, Ibadan	Oyo	128
3	Fagbesa Market, Osogbo	Osun	48
4	Okelewo Market, Abeokuta	Ogun	78
5	Ayo Fayose Market, Ado-Ekiti	Ekiti	38
6	Olukayode Shopping Complex, Akure	Ondo	53
	**Total**		**600**

### 2.2 Measurement of construct

Social motivation was measured using the McClelland's human motivation theory which consists of power, affiliation and achievement needs. This was drafted from a questionnaire study of García-Rodríguez et al. (2013).

## 3 Results

Table is a distribution of the respondent's demographics characteristics. It depicts the respondent's gender, age, age of business, reasons for setting up business, additional qualifications, educational qualifications, presence of technical skill, income level and parents income level during engagement in informal electronics business.

Table 2 presents the statistical results of the demographic characteristics of respondents in the six states used for this study. A total of 435 (80%) male respondents and 109 (20%) female respondents were included in the study. Out of the 435 male respondents, 109 (34.9%) were from Lagos State, 59 (10.8%) are from Ogun State, 91(16.7%) from Oyo State, 42 (7.7%) from Osun State, 26 (4.8%) from Ondo State and 27 (5%) from Ekiti State while the female respondents were 19 (3.5%) from Lagos State, 9 (1.7%) from Ogun State, 37(6.8%) were from Oyo State, 6 (1.1%) from Osun State, 27 (5%) were from Ondo State and Ekiti State had 11(2%) respondents. This implies that there are more male than female respondents across the selected informal electronics markets in southwest, Nigeria. The result from Ondo State proved otherwise in the analyses with higher distribution from the female gender.

**Table 2:** Demographic information of informal entrepreneurs in six selected electronics markets across the southwest states, Nigeria.

Demographic Variables		Lagos State	Ogun State	Oyo State	Osun State	Ondo State	Ekiti State	Total
Gender	Male	190	59	91	42	26	27	**435**
	Female	19	9	37	6	27	11	**109**
Marital Status	Single	108	39	46	26	33	14	**266**
	Married	95	27	51	17	20	17	**227**
	Others	6	2	31	5	0	7	**51**
Age of Business	Less than 5 years	70	30	23	15	22	11	**171**
	5–9 years	63	14	43	16	17	8	**161**
	10–14 years	46	16	35	9	8	15	**129**
	15 years - above	30	8	27	8	6	4	**83**

### 3.1 Marital status

The marital status distribution of the respondents as shown in the Table 2 revealed that 266 (48.9%) respondents in the study were singles and this varies with Lagos State, Ogun State, Oyo State, Osun State, Ondo State and Ekiti State having 108 (19.9%), 39 (7.2%), 46 (8.5%), 26 (4.8%), 33 (6.1%), 14 (2.6%) respectively while 95(17.5%), 27 (5%), 51 (9.4%), 17 (3.1%), 20 (3.7%), 17 (3.1%) correspondingly were for Lagos State, Ogun State, Oyo State, Osun State, Ondo State and Ekiti State with 227 married respondents (41.7%). The others in the marital status include those divorced and widowed and the respondents were tagged with 6 (1.1%), 2 (0.4%), 31 (5.7%), 5 (0.9%), 0, 7 (1.3%) for Lagos State, Ogun State, Oyo State, Osun State, Ondo State and Ekiti State with 51 (9.4%) in turn. This inferred that most of the respondents that ventured into informal electronics business were singles and thus were trying to make meanings with their lives before venturing into another phase.

### 3.2 Age of business

Considering the number of years, that the respondent's business has being in operation, it was revealed from the Table 2 that 171 respondents having less than 5 years of business age was computed 70 (12.9%), 30 (5.5%), 23 (4.2%), 15 (2.8%), 22 (4%), 11 (2%) for Lagos State, Ogun State, Oyo State, Osun State, Ondo State and Ekiti State respectively. The age band of 5–9 years for the business age reflected 161(29.6%) respondents with 63 (11.6%), 14 (2.6%), 43 (7.9%), 16 (2.9%), 17 (3.1%),8 (1.5%) for Lagos State, Ogun State, Oyo State, Osun State, Ondo State and Ekiti State correspondingly while Lagos State, Ogun State, Oyo State, Osun State, Ondo State and Ekiti State were disparately represented with 46 (8.5%), 16 (2.9%), 35 (6.4%), 9 (1.7%), 8 (1.5%) and 15(2.8%) totalling 129 (23.7%) for the age band of 10–14 years. For 15 years and above, the table presented 83 (15.3%) comprising of 30 (5.5%), 8 (1.5%), 27 (5%), 8 (1.5%), 6 (1.1%), 4 (0.7%) depicting the respondents' rates from Lagos State, Ogun State, Oyo State, Osun State, Ondo State and Ekiti State. The inference from the age band of the business is less than 5 years with a clear depiction of 171 (31.4%) of newly entrance into the market viz-a-viz a quick exit.

### 3.3 Descriptive statistics of responses based on the state of the informal entrepreneurs

The following table below shows the descriptive statistics of responses from the informal entrepreneurs in the electronics markets in southwest, Nigeria established on their respective State of business engagement. The relevance of showing the descriptive statistics is to identify the degree of importance from their states in the electronics informal market in lieu with the respective measures (see Table 3).

**Table 3:** Descriptive statistic of responses based on the state of informal entrepreneurs on social motivation.

	Item		LG	OG	OY	OS	OD	EK
1	I do this job because I want to create social value for myself (M_s_)	M	4.2010	4.2647	3.4609	3.5625	2.9434	3.6579
		SD	1.07766	.87447	1.01874	.61562	.94899	.78072
2	There is probability that this informal business will enable me to enhance my social status. (P_s_)	M	4.2488	4.2059	3.5156	3.7292	3.5094	.75053
		SD	.84655	.82061	1.07940	.67602	.79958	.80891
3	Informal business provides the required platform for social incentives such as catering for self and family as well as cordial relationship (I_s_)	M	4.3206	4.0882	3.4687	3.7083	3.1698	3.6842
		SD	.82486	1.03282	.96341	.71335	.75284	82329
4	I maintain my informal business operation because I want to preserve my interpersonal relationships with my colleagues	M	3.8421	3.9265	3.2187	3.3542	3.2642	3.3947
		SD	1.03262	1.11055	.87788	.63546	.83553	.75101
5	My involvement in the informal market enables me to establish, maintain, and restore positive affective relations with others.	M	3.9809	4.0147	3.4531	3.6875	3.4151	3.7632
		SD	.93006	1.04371	.98700	.85443	.77046	.80052
6	Cordial relation with other informal entrepreneurs encourages my willingness to operate informally.	M	3.9952	3.8235	3.6406	3.8333	3.2264	3.8158
		SD	.90669	1.10550	1.05543	.83369	.86916	.86005
7	I love this job because it enables me to have strong effects on other people	M	3.9043	4.0147	3.5469	3.6667	3.3585	3.7368
		SD	1.14372	.96958	.96277	.78098	.96266	.88932
8	I participate in this business because it is a means of influencing the behavior of another person	M	3.5167	3.6765	3.4922	3.8125	3.3019	3.4211
		SD	1.18117	1.13875	1.12240	.86679	.82240	1.05739
9	This business helps me in maintaining my position as the breadwinner of my home	M	4.1531	3.9706	3.6875	3.7292	3.3962	3.7368
		SD	.98815	1.03622	1.16206	.98369	.92694	3.6316
	**Social Motivation**	M	4.0181	3.9984	3.4983	3.6759	3.2872	3.6491
		SD	**.60675**	.66014	**.76942**	.56602	**.39790**	.63305

Source: Field Survey Result (2018).

In general, Table 3 statistical result displayed that respondents from the various states agree with the measures of social motivation contained in the survey. This was clearly displayed from the states of the respondents reflecting a mean of 4.0181, 3.9984, 3.4983, 3.6759, 3.2872 and 3.6491 for Lagos state, Ogun state, Oyo state, Osun state, Ondo state and Ekiti state respectively. From the above mean score, it can be postulated that the respondents agree that the respective states, where the informal market are. links with the perceived level of social motivation. This was clearly displayed with the highest mean score of 4.0181 coming from Lagos State while the lowest mean score was from the Ondo state respondents with the mean score of 3.2872. It can be posited from the displayed results that the respective States indicates the environmental influence from the view point of social encouragement the respondents get from engaging in the informal business in these respective states and this was reflected in Lagos state, being the economic hub of Nigeria.

### 3.4 Test of hypothesis

The study hypothesis wads stated as:

H_a1_ - Social motivation is a significant predictor of sustainable competitive advantage in the informal market.

The hypothesis was statistically tested using linear regression to: (i) identify whether or not there is a relationship; (ii) examine the degree of the relationship, between the independent variable (that is, the social motivation) and dependent variable (sustainable competitive advantage); (iii) to access the predictor importance of the variable and finally (iv) to analyse the significance effect of the variable under study.

The model summary (see Table 4) shows how much of the variance of the dependent variable (sustainable competitive advantage) is explained by the independent variable (social motivation). In this case, the R square shows a coefficient determination of about 0.151 and if expressed by a percentage will be 15.1%. This infers that 15.1% variation of sustainable competitive advantage is predicted by the measures of social motivation. The findings are supported by Analysis of Variance ANOVA (F test) results that the model or none of the parameters was equal to Zero (see Table 5).

**Table 4:** Model summary.

Model	R	R Square	Adjusted R Square	Std. Error of the Estimate
1	0.389^a^	0.151	0.150	0.77200

Source: Field Survey Result (2018).

a Predictors: (Constant), Social Motivation

**Table 5:** ANOVA^a^.

Model		Sum of Squares	df	Mean Square	F	Sig.
1	Regression	57.501	1	57.501	96.480	0.000^b^
	Residual	323.026	542	0.596		
	Total	380.528	543			

The ANOVA table shows that the F value is 96.480 at .000^b^ significance level. The implication is that social motivation has a significant effect on sustainable competitive advantage.

a Dependent Variable: Sustainable competitive advantage.

b Predictors: (Constant), Social Motivation.

The coefficient table above depicts the statistically significant contribution reflected in the simple model expressing the extent to which variables included in the model contributed to the prediction of the dependent variable via the viewing of the sig column in the table. The level of significance was based on a level of 0.05 for a two-tail test, with the absolute value of the test statistics (t) greater than or equal to the critical value of 1.96. The model revealed that social motivation has a statistical significance in predicting sustainable competitive advantage recording the beta value of (*beta* = 0.389 with t-val (9.822) higher than 1.96, sig.000 p < .05) (see Table 6). The confidence level of the mean score of the sustainable competitive advantage for social motivation in Southwest, Nigeria at the 99% falls between 4.0667 and 4.2891 while at the 95% level of confidence was at 4.0934 and 4.2624. This means that social motivation makes a strong unique contribution to explaining sustainable competitive advantage in the informal market. This implies that for each unit increase in social motivation there is up to 0.389 unit increase in sustainable competitive advantage.

**Table 6:** Coefficient Table for the independent variable (Social Motivation).

Model		Unstandardized Coefficients		Standardized Coefficients	t	Sig.
		B	Std. Error	Beta		
1	(Constant)	2.591	0.156		16.639	0.000
	Social	0.391	0.040	.389	9.822	0.000

Source: Field Survey Result (2018).

### 3.5 Decision

The significance level below 0.05 implies a statistical confidence of above 95%. This implies that the social motivation has an effect on sustainable competitive advantage. Thus, the null hypothesis (H_01_) was rejected, while the alternate hypothesis (H_a1_) which posits that social motivation is a significant predictor of sustainable competitive advantage in informal market was accepted. Therefore, it can be linked that the Marcia theory of identity achievement relates those two distinct parts form an adolescent's identity: crisis and commitment to the social drive of achievement was a necessity in the niche of competition amongst the operators in the informal market. This acts as a need for the sustainment of the activity in the informal market as against the rules set to bare the ongoing activities by relating the informal entrepreneurs' complexities of engagement in the electronics market to their respective status identity or vice-versa. As this as been the case of the Nigerian youth at getting needs met even when a dire exists as against the expected African cultural heritage of value and goals attainment. We can therefore ascertain that the Marcia theory shows how the attainment of success on the global stage is inclined to correct the irony of age with success.

## 4 Conclusion

The study has revealed that social motivation characterised by different social dynamics has a positive significant effect on sustainable competitive advantage in the informal market. The study reflected that social motivation makes a strong contribution to explaining sustainable competitive advantage in the informal market. The implication of this result is that sustainable competitive advantage of the informal entrepreneurs in the electronics market is socially motivated. This finding is in agreement with the works of Badenhorst-Weiss et al. (2014) who posit that sustainable formal independent small businesses are socially motivated into the business activities while retaining their competitive nature via the respective differentiation made. Moreover, Block's et al. (2014) position on necessity entrepreneurs being more likely to pursue a cost leadership strategy than a differentiation strategy as a result of socioeconomic attributes agrees with the study's findings.

On this note, this study has provided new insights on the social motivation of informal entrepreneurship in a developing country like Nigeria. It has provided empirical evidences on the effect of social motivation on business performance. The uniqueness of each Nigerian Southwestern state as regards the social motivations of informal entrepreneurship was brought to the fore. It was discovered as the creation for social value is the backbone for the commitment in the informal market with a high level of independence from the low cost of engaging in the business activity as other required setup costs are averted or slightly overcome through the engagement of youthful energy.

## Declarations

### Author contribution statement

Adebanji William Ayeni: Analyzed and interpreted the data; Wrote the paper.

Olaleke Ogunnaike and Oluwole Iyiola: Conceived and designed the experiments; Analyzed and interpreted the data; Wrote the paper.

Edidiong Ayeni: Performed the experiments; Wrote the paper.

### Funding statement

This research did not receive any specific grant from funding agencies in the public, commercial, or not-for-profit sectors.

### Data availability statement

Data will be made available on request.

### Declaration of interests statement

The authors declare no conflict of interest.

### Additional information

No additional information is available for this paper.

## Appendix A Supplementary data

Ifemi Supplementary File Ifemi Supplementary File
