# Supplementary material for: Harnessing the sustainable competitive advantage of social motivation in the informal market: A West African society insight
Source: Heliyon. 2021 Jul 10;7(7):e07538. doi: 10.1016/j.heliyon.2021.e07538 (PMC8287236; doi:10.1016/j.heliyon.2021.e07538)
Supplement: Ifemi Supplementary File [file mmc1.docx]

**Appendix**

**Measurement of Construct**

| Constructs | Variables | No of Items | Sources |
| --- | --- | --- | --- |
| Motivations | Social Motivation | 9 | García-Rodríguez, Gil-Soto, Ruiz-Rosa and Sene (2013); Williams and Nadin (2010) |

| **KMO and Bartlett's Test** | | |
| --- | --- | --- |
| Kaiser-Meyer-Olkin Measure of Sampling Adequacy. | | .910 |
| Bartlett's Test of Sphericity | Approx. Chi-Square | 8753.425 |
|  | df | 496 |
|  | Sig. | .000 |

| **Communalities** | | |
| --- | --- | --- |
|  | Initial | Extraction |
| Social Value | 1.000 | .303 |
| Social Status | 1.000 | .342 |
| Social Incentives | 1.000 | .432 |
| Interpersonal Relationship | 1.000 | .293 |
| Affective Relations | 1.000 | .422 |
| Cordial Relation | 1.000 | .402 |
| Strong effects | 1.000 | .348 |
| Influencing the behaviour of others | 1.000 | .303 |
| Breadwinner Position Maintaince | 1.000 | .350 |
| Extraction Method: Principal Component Analysis. | | |

| **Total Variance Explained** | | | | | | |
| --- | --- | --- | --- | --- | --- | --- |
| Component | Initial Eigenvalues | | | Extraction Sums of Squared Loadings | | |
|  | Total | % of Variance | Cumulative % | Total | % of Variance | Cumulative % |
| 1 | 9.115 | 28.483 | 28.483 | 9.115 | 28.483 | 28.483 |
| 2 | 4.944 | 15.451 | 43.934 | 4.944 | 15.451 | 43.934 |
| 3 | 2.099 | 6.559 | 50.493 |  |  |  |
| 4 | 1.200 | 3.751 | 54.245 |  |  |  |
| 5 | 1.091 | 3.409 | 57.653 |  |  |  |
| 6 | 1.056 | 3.299 | 60.953 |  |  |  |
| 7 | .899 | 2.811 | 63.764 |  |  |  |
| 8 | .833 | 2.602 | 66.366 |  |  |  |
| 9 | .824 | 2.576 | 68.941 |  |  |  |
| Extraction Method: Principal Component Analysis. | | | | | | |

**Motivation Descriptive Statistics**

|  | | | |
| --- | --- | --- | --- |
|  | N | Mean | Std. Deviation |
| SM | 544 | 3.7661 | .68965 |
| Valid N (listwise) | 544 |  |  |

**Confidence Indicator at 95%**

| **Case Processing Summary** | | | | | | |
| --- | --- | --- | --- | --- | --- | --- |
|  | Cases | | | | | |
|  | Valid | | Missing | | Total | |
|  | N | Percent | N | Percent | N | Percent |
| Business Performance (Sustainable Competitive Advantage) | 541 | 99.4% | 3 | 0.6% | 544 | 100.0% |

| **Descriptives** | | | | |
| --- | --- | --- | --- | --- |
|  | | | Statistic | Std. Error |
| Business Performance (Sustainable Competitive Advantage) | Mean | | 4.1779 | .04301 |
|  | 95% Confidence Interval for Mean | Lower Bound | 4.0934 |  |
|  |  | Upper Bound | 4.2624 |  |
|  | 5% Trimmed Mean | | 4.2177 |  |
|  | Median | | 4.2500 |  |
|  | Variance | | 1.001 |  |
|  | Std. Deviation | | 1.00041 |  |
|  | Minimum | | 1.00 |  |
|  | Maximum | | 17.50 |  |
|  | Range | | 16.50 |  |
|  | Interquartile Range | | 1.25 |  |
|  | Skewness | | 3.853 | .105 |
|  | Kurtosis | | 57.395 | .210 |

**Confidence Indicator at 99%**

| **Case Processing Summary** | | | | | | |
| --- | --- | --- | --- | --- | --- | --- |
|  | | | | | | |
|  | Cases | | | | | |
|  | Valid | | Missing | | Total | |
|  | N | Percent | N | Percent | N | Percent |
| Business Performance (Sustainable Competitive Advantage) | 541 | 99.4% | 3 | 0.6% | 544 | 100.0% |

| **Descriptives** | | | | |
| --- | --- | --- | --- | --- |
|  | | | Statistic | Std. Error |
| Business Performance (Sustainable Competitive Advantage) | Mean | | 4.1779 | .04301 |
|  | 99% Confidence Interval for Mean | Lower Bound | 4.0667 |  |
|  |  | Upper Bound | 4.2891 |  |
|  | 5% Trimmed Mean | | 4.2177 |  |
|  | Median | | 4.2500 |  |
|  | Variance | | 1.001 |  |
|  | Std. Deviation | | 1.00041 |  |
|  | Minimum | | 1.00 |  |
|  | Maximum | | 17.50 |  |
|  | Range | | 16.50 |  |
|  | Interquartile Range | | 1.25 |  |
|  | Skewness | | 3.853 | .105 |
|  | Kurtosis | | 57.395 | .210 |
